# Multispectral analysis-ready satellite data for three East African mountain ecosystems

**DOI:** 10.1038/s41597-024-03283-3

**Published:** 2024-05-09

**Authors:** Netra Bhandari, Lisa Bald, Luise Wraase, Dirk Zeuss

**Affiliations:** Department of Geography, Environmental Informatics, Philipps-Universität Marburg, Deutschhausstrasse 12, 35032 Marburg, Germany

**Keywords:** Environmental sciences, Ecology

## Abstract

The East African mountain ecosystems are facing increasing threats due to global change, putting their unique socio-ecological systems at risk. To monitor and understand these changes, researchers and stakeholders require accessible analysis-ready remote sensing data. Although satellite data is available for many applications, it often lacks accurate geometric orientation and has extensive cloud cover. This can generate misleading results and make it unreliable for time-series analysis. Therefore, it needs comprehensive processing before usage, which encompasses multi-step operations, requiring large computational and storage capacities, as well as expert knowledge. Here, we provide high-quality, atmospherically corrected, and cloud-free analysis-ready Sentinel-2 imagery for the Bale Mountains (Ethiopia), Mounts Kilimanjaro and Meru (Tanzania) ecosystems in East Africa. Our dataset ranges from 2017 to 2021 and is provided as monthly and annual aggregated products together with 24 spectral indices. Our dataset enables researchers and stakeholders to conduct immediate and impactful analyses. These applications can include vegetation mapping, wildlife habitat assessment, land cover change detection, ecosystem monitoring, and climate change research.

## Background & Summary

**Fig. 1:**
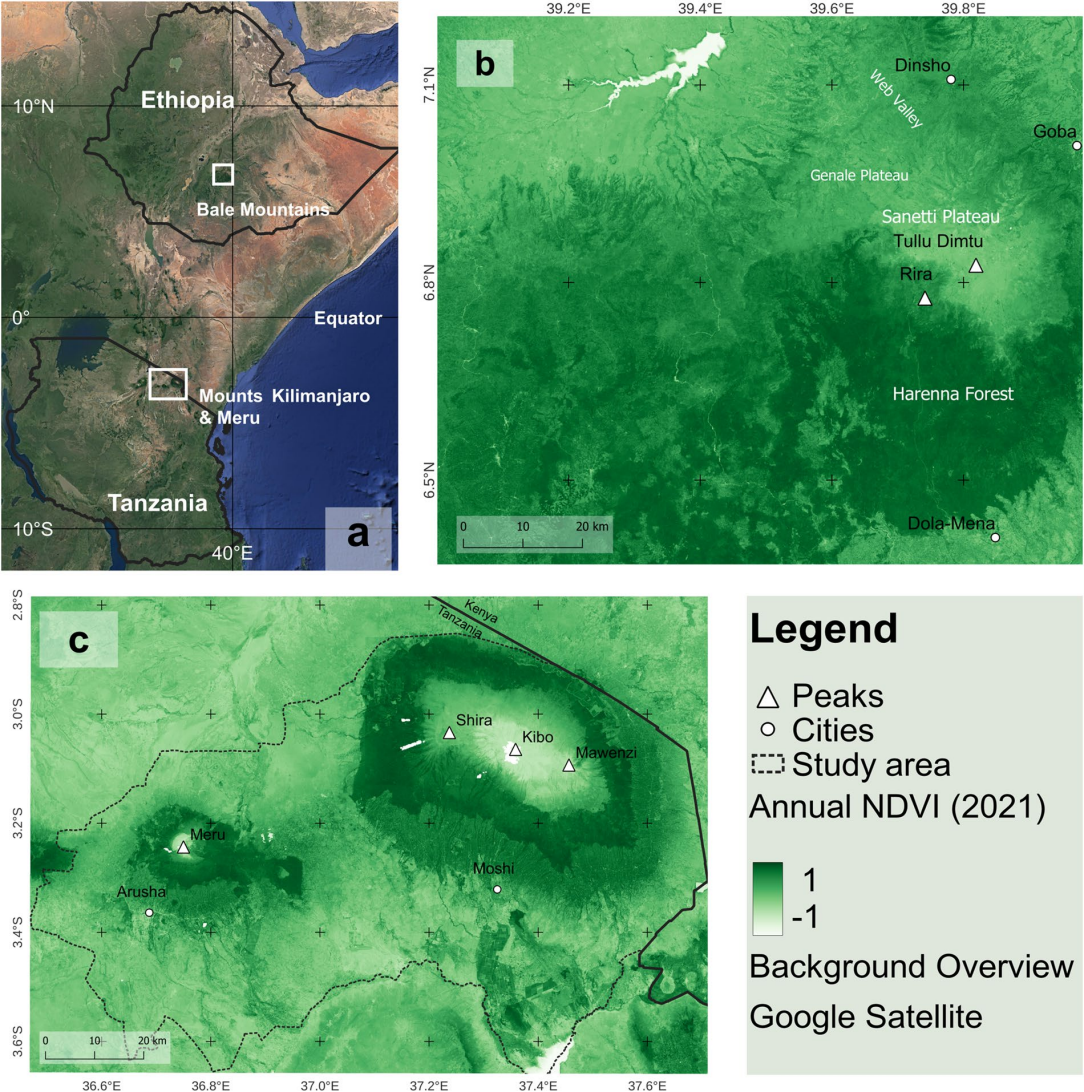
Mountains in East Africa covered by our dataset: (**a**) Overview. (**b**) Bale Mountains (Ethiopia), (**c**) Mounts Kilimanjaro and Meru (Tanzania) ecosystem. The Normalized Difference Vegetation Index (NDVI) provided with our dataset is shown as an example.

Mountain ecosystems are increasingly being affected by climate and land use changes, population growth, pollution, exotic species introduction, and rural exodus^1^. These drivers of change impact biodiversity as well as the millions of people who live in these ecosystems^2^. The mountains of East Africa are hotspots of biodiversity and support the livelihoods of millions of people^1^. It is crucial to consider the changes occuring in these regions as they provide multiple ecosystem services. These services include food, fodder, timber, fuelwood (provisioning services), climate regulation, soil formation and protection, pollination and pest regulation, hazard regulation (regulating services), aesthetic and recreation services and functions (cultural services)^2^. However, compared to other terrestrial ecosystems, research on different ecosystem services provided by mountains is not extensive enough^2^. This study focuses on three East African mountain ecosystems namely Bale Mountains (Ethiopia), Mounts Kilimanjaro and Meru (Tanzania; Fig. 1).

The Bale Mountains of Ethiopia support a remarkable diversity of endemic flora and fauna such as the Ethiopian wolf (*Canis simensis*), the Mountain Nyala (*Tragelaphus buxtoni*)^3^, and plants such as *Lobelia* and *Senecio* species. These mountains feature a steep elevational gradient of vegetation starting from the top with afro-alpine dwarf shrubland (3 800–4 377 m), an Ericaceous belt (3, 200–3,600 m), upper and lower afro-montane forests including *Bambusa* forest, *Juniperus*, *Hypericum* and *Hygenia* woodland descending to Anthropocene-influenced areas of farmland and settlements (2,000–3,400 m)^4,5^. The rainforests in the south also host wild coffee (*Coffea arabica*). These vegetation gradients create distinct ecological zones at varying altitudes which support a wide range of species including many endemics^5^. The Bale Mountains also supply multiple ecosystem services such as freshwater regulation for lowland areas, food supply, medicinal plants^6^, and habitat provision for the endemic fauna (e.g. *Tachyoryctes macrocephalus*^3^) to name a few. Notably, these mountains encompass around 25% of all afro-alpine and afro-montane forest habitats in Africa^7–9^. However, they face increasing threats from habitat loss and fragmentation^10^, primarily driven by population growth^10^, climate change^11^, agricultural expansion^12^, and political-religious unrest^13^. These factors critically impact biodiversity, disrupting ecological balances and species migration, reducing genetic diversity, and undermining essential ecosystem services^13^. Compounding these issues, the northwestern part of the highlands has seen an increase in human settlements and livestock with approximately 1,449 permanent and 3,143 seasonal residents, along with their cows, sheep, goats and also some horses, mules and donkeys^14^. This increase in human activity places further pressure on the already fragile environment, underscoring the complex challenges in conserving and sustainably managing the Bale Mountains^14^.

With an elevation ranging from 700 m to 5,895 m above sea level, Mount Kilimanjaro is the tallest peak in Africa and the highest free-standing mountain in the world. This mountain encompasses forest and wildlife reserves, as well as local agricultural areas which represents a unique range of climatic and vegetation zones from cloud forests and alpine vegetation with *Helichrysum* species at around 4,500 m^15^ to hot savannas at the foothills. Since 1973, ecosystems above 2,700 m have been protected in the Kilimanjaro National Park and since 2006, areas above 1,800 m have also been included^16^. The mountain provides freshwater for major river systems^15^ and is home to numerous species, many of which are threatened^17^. Many threats to its natural ecosystems and biodiversity, such as increasing population pressure leading to increased demands for freshwater, grazing, and land have been recognized and are actively being researched^18^. In the past, major land use changes occurred in the foothills between 1979 and 2000, when the diverse savanna was converted to agricultural land^19^. The Kilimanjaro region hosts around 1.8 million people^20^ largely involved in farming and tourism. The majority of people live in Chagga home gardens^15,18^ and grow crops like maize, beans, and bananas^18^. The region also has a significant portion of land cultivated for commercial coffee plantations^17^.

Mount Meru the second-highest peak (4,566 m) in Tanzania, is surrounded by several protected areas. The mountain harbors 13 habitat types dominated by *Croton-Calodedrum* forests in the submontane regions, *Cassipurea* forests in the mid-altitude regions, and *Juniperus* forests in the higher altitude regions^19^. The fauna is also rich in medium-sized carnivores such as cheetah (*Acinonyx jubatus jubatus*), leopard (*Panthera pardus*), spotted hyena (*Crocuta crocuta*), aardwolf (*Proteles cristata*), black-backed jackal (*Lupulella mesomelas*), bat-eared fox (*Otocyon megalotis*), African wild dog (*Lycaon pictus*), and small mammals such as rodents and shrews^21,22^. Mount Meru is also a recharge zone, providing fresh water to the Arusha region in the foothills^23^. Landsat imagery has shown that forest bridges between Mounts Kilimanjaro and Meru once served as a corridor for arthropod dispersal and mammal and reptile migration, but these biogeographically important bridges have disappeared over time due to human settlement and agricultural expansion^19,24^. The Arusha region which encompasses Mount Meru has a population of around 2.3 million^20^. The people in the region of Mount Meru largely grow coffee and banana^25^.

To address the various above-mentioned challenges in East African mountains at a landscape scale, researchers and stakeholders need easy and quick access to analysis-ready remote sensing data. Here the term analysis-ready remote sensing data refers to data that has been made spatially seamless, contains minimal cloud cover, has undergone atmospheric correction and geometric alignment, and is therefore ready for further analysis without the need for additional processing.

Typically, satellite data are freely available and easy to download using sources such as the Copernicus Open Access Hub (https://scihub.copernicus.eu/) from the European Space Agency (ESA) or Earth Data from the National Aeronautics and Space Administration (NASA; https://www.earthdata.nasa.gov/). While satellite data can be easily downloaded, finding high-quality images usually is time-consuming, especially for East African mountains, which have high to complete cloud cover for many months^26,27^. The uniform processing of temporal satellite data is also resource-intensive and requires expert knowledge, computing power, and storage capacity to convert the raw data into analysis-ready information. Moreover, obtaining high-quality data is not always straightforward, as limited power and internet access in remote or under-resourced areas can cause significant delays or even prevent access to high-quality data altogether. These limitations can slow down progress in research and monitoring of valuable socio-ecological systems, posing a significant challenge for scientists and land managers. Both ESA and NASA currently offer analysis-ready data products. NASA presently provides the Harmonized Landsat Sentinel-2 (HLS)^28^ product, featuring a spatial resolution of 30 meters. Concurrently, ESA has planned to release two important products: Sentinel-2 Level-2H and Sentinel-2 Level-2F^29^. These datasets will encompass harmonized and fused data from Sentinel-2 and Landsat sources, respectively. Despite these advancements, the specific needs of research in the East African mountains like the Bale Mountains, and Mounts Kilimanjaro and Meru, which have been areas of extensive study for over two decades. They call for a dedicated analysis-ready data product at a spatial resolution of 10 m, which is valuable for the various stakeholders.

Since their launch in 2015 and 2017, the two Sentinel-2 satellites^30^ (Sentinel-2A and 2B) have become a popular choice for various applications such as vegetation mapping, wildlife habitat assessment, and land cover change detection, as they provide satellite imagery with 10 m spatial resolution and a wide range of multispectral channels. However, Rufin *et al*.^31^ highlighted that there is a problem of multitemporal inconsistency between the two Sentinel-2 satellites and a problem of geometric misalignment of up to 14 m (i.e. more than one Sentinel-2 pixel of 10 m resolution) between Landsat and Sentinel-2 satellite images. Such inconsistencies in a single-sensor time series as well as time series from multiple sensors can hinder the process of understanding long-term changes at the landscape scale.

To overcome this problem, Frantz^32^ developed a free-of-charge and open-source software called *Framework for Operational Radiometric Correction of Environmental Monitoring* (*FORCE*; https://github.com/davidfrantz/force). *FORCE* adheres to the data processing standards stipulated by the Committee on Earth Observation Satellites (https://ceos.org/ard/), ensuring the delivery of analysis-ready data that conforms to widely accepted community-agreed standards. *FORCE* is a solution for downloading and processing multiple Sentinel-2 images and for providing spatially seamless, nearly cloud-free, atmospherically corrected analysis-ready data^32^. It can also solve the problem of geometric misalignment between sensors in time-series data and can be used to generate higher-level products such as spectral indices^31,32^. However, processing with *FORCE* has some limitations, as explicit knowledge is required to work with the software and processing and familiarizing oneself with the software might take a long time. Moreover, *FORCE* is only available as a command line software for the Linux operating system, and due to the lack of a graphical user interface, the program may not be easily accessible for all parties for whom it might be beneficial. Furthermore, it has large storage and processing needs with around 6 TB storage space necessary for the dataset provided here, which was processed using 12 physical CPU cores and 62 GB random access memory.

Despite these challenges, we have successfully utilized *FORCE* to process and provide atmospherically corrected and geometrically aligned analysis-ready Sentinel-2 satellite imagery processed with *FORCE* for the timeframe of 2017 to 2021 for three mountains in East Africa: Bale Mountains in Ethiopia and Mounts Kilimanjaro and Meru in Tanzania. The dataset is provided at 10 m spatial resolution and at several temporal resolutions: images for each year from 2017 to 2021 and monthly images for each month from January 2017 to December 2021. In addition to the multispectral Sentinel-2 bands (e.g. red, green, blue, near-infrared), spectral indices are also provided, for example, the Normalized Difference Vegetation Index (NDVI)^33^, the Enhanced Vegetation Index^34^ or the Normalized Difference Water Index^35^. In total 1.08 TB of analysis-ready satellite data for Bale Mountains and 1.94 TB for Mounts Kilimanjaro and Meru are provided.

The dataset has broad applicability for researchers and stakeholders such as government officials, protected areas managers, and nature conservationists. It can be used for species distribution modeling, the generation of monthly and annual weather maps from available but limited weather stations, the upscaling of ecosystem services from local to a landscape scale, vegetation mapping, wildlife habitat assessment, land cover change detection, ecosystem monitoring, and climate change research. Previously, high-quality satellite datasets produced with *FORCE* have been successfully used for example, to map grassland mowing events^36^, drought in Germany^37^, and to map crop types and cropping systems in Nigeria^38^.

## Methods

We created analysis-ready multispectral data, with imagery from two different satellite systems. The Sentinel-2^30^ data provided in this study were recorded by the two identical Sentinel-2 satellites (Sentinel-2A and Sentinel-2B). Images from the Landsat^39^ 8 and 9 satellites were used to align the images from the two Sentinel satellites. Landsat provides images with a spatial resolution of 30 m but with a lower spectral resolution than Sentinel-2. A higher spectral resolution is important for our study as it allows for the detection of a wider range of wavelengths, leading to more detailed and accurate identification of surface materials and features. This enhanced resolution provides greater discrimination capabilities in various applications, such as forest disturbances^40^ or forest canopy properties^41^. As the Landsat satellites do not have a problem of correct geometric alignment, they can be used to correct the position of the Sentinel-2 data. Processing satellite remote sensing data involves distinct processing levels (https://force-eo.readthedocs.io/en/latest/howto/l2-ard.html). Level 0 data is acquired at the satellite and is typically unavailable to end-users. Level 1 data, like Sentinel-2 L1C data, undergoes radiometric correction and georectification, which are essential processes to adjust for sensor and atmospheric inaccuracies and to geometrically refine the imagery before making it available to users. At level 2, additional corrections, such as atmospheric or topographic adjustments, are applied, such as for Sentinel-2 L2A data, to eliminate distortions in the imagery caused by atmospheric conditions and terrain variations. Level 3 data comprises level 2 data that has undergone either temporal or statistical aggregation, and the dataset presented in this study falls into the level 3 category. Processing to level 3 is necessary to provide the user with analysis-ready data.

### Level 1 data acquisition

The Sentinel-2 images for the years 2017 to 2021 were downloaded from the European Space Agency hub at L1C for the three mountain ecosystems using the software *FORCE*^32^ (version 3.7.10). The images were partitioned into tiles, following a standardized system and naming convention. For the Bale Mountains, Sentinel-2 data with tile number T37NEH (364 images in total) were downloaded, while for Mounts Kilimanjaro and Meru, data with tile numbers T37MCS and T37MBS (548 images in total) were obtained.

**Fig. 2:**
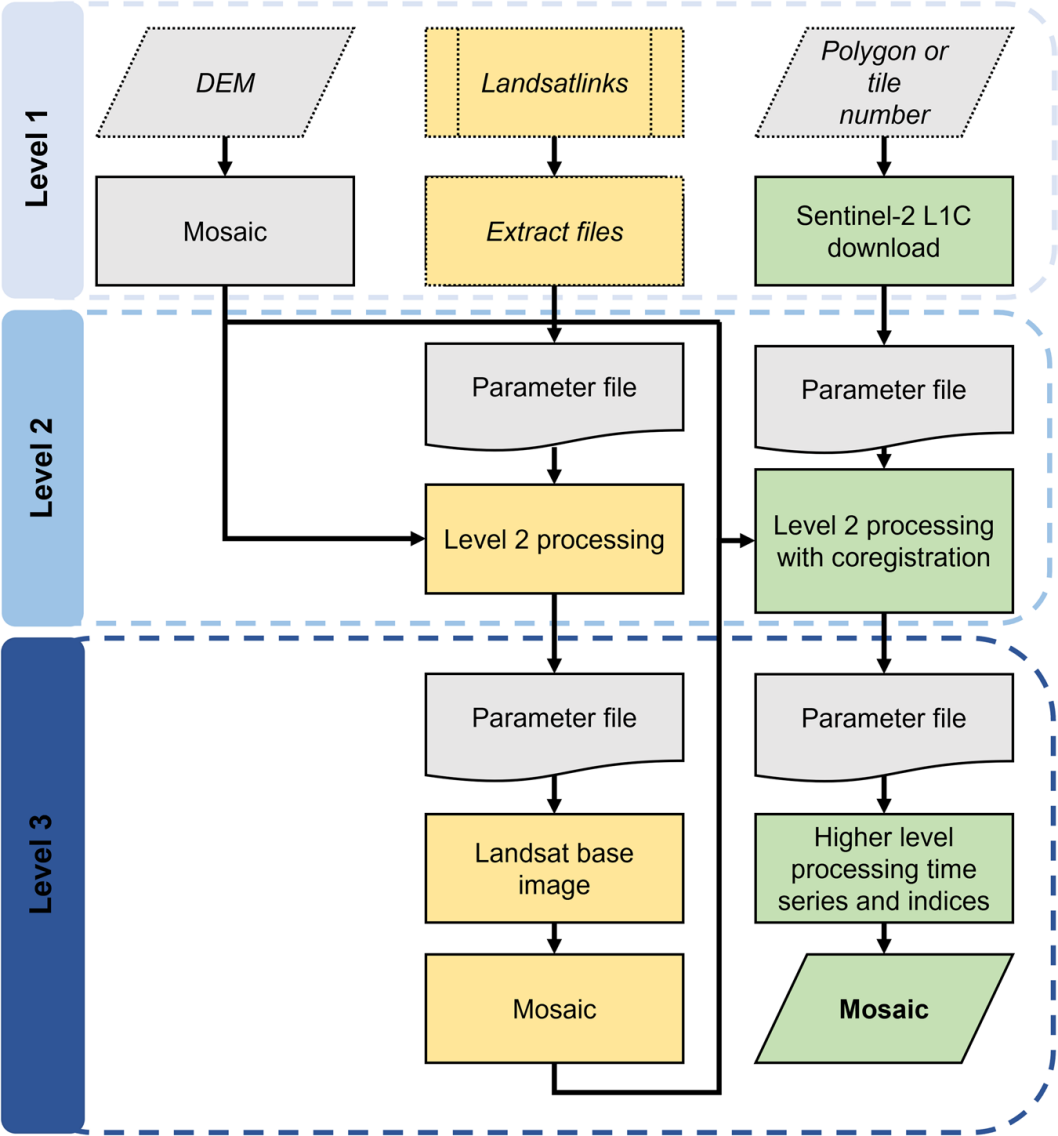
Workflow: We present here a simplified overview of the workflow used to create our dataset. The data that were downloaded or processed using *FORCE* are indicated by a solid line, while those that were downloaded or processed without the use of *FORCE* are indicated by a dotted line. The processing is divided into three parts: *FORCE* processing level 1, contains the steps for downloading the Sentinel-2 (L1C; green) and the corresponding Landsat (L1TP; yellow) tiles using an area polygon or the tile number of the respective satellite product. The Landsat data were retrieved by the software *Landsatlinks*. The digital elevation model (DEM) tiles were mosaiced into a virtual raster to be used for atmospheric correction in the *FORCE* level 2 processing. At *FORCE* processing level 2, Landsat and Sentinel-2 tiles were processed separately in sensor-specific steps: first, all Landsat tiles were processed. In the second step, all Landsat images were processed into a Landsat base image (*FORCE* level 3 product). Third, the Sentinel tiles were processed and coregistered using the Landsat base image as a reference. At level 3, the level 2 Sentinel (corresponds to European Space Agency’s Sentinel-2 L2A data) tiles were further processed into time series products and multispectral indices.

Landsat data fully covering the above-mentioned Sentinel-2 tiles and consisting of Landsat 8 and 9 imagery were downloaded for the years 2013 to 2021 (Ethiopia: 685 images, Tanzania: 733 images) at L1TP using the software *Landsatlinks* (version 1.0.0; https://github.com/ernstste/landsatlinks; Fig. 2). *Landsatlinks* provides users with a command line interface to retrieve download URLs for Landsat data with a machine-to-machine Application Programming Interface. The files were then downloaded from NASA Earth Data and extracted via the command line using a list of the generated Landsat download links.

Both Sentinel-2 and Landsat 8 and 9 datasets were downloaded in the World Geodetic System projections (WGS84) Universal Transverse Mercator (UTM) zone 37N for Bale Mountains and UTM zone 37 S for Mounts Kilimanjaro and Meru.

To perform a topographical correction of the Sentinel-2 and Landsat data a digital elevation model was needed (Fig. 2). We used the Copernicus Global Digital Elevation Models^42^ of 30 m resolution for Bale Mountains, Mount Kilimanjaro and Meru. The digital elevation model images were downloaded from Open Topography (https://portal.opentopography.org).

### Landsat level 2 processing and generation of a base image

To use Landsat data to correct the position of Sentinel-2 data, the former must be processed first. Each Landsat image was processed using cloud detection and atmospheric correction (including topographic correction, adjacency effect correction, Bidirectional Reflectance Distribution Function correction, and multiple scattering correction)^32^. Topographic correction is vital in mountainous regions to adjust for varying solar irradiance and angles, reducing misclassification in remote sensing data due to terrain-induced variations in reflectance and shadow effects^43^. This correction is primarily achieved using empirical methods and digital elevation models^43^. Furthermore, atmospheric correction of satellite images is a processing step that significantly reduces or eliminates the influence of the atmosphere on the imagery^44^. This ensures that the data more accurately represents the actual surface characteristics^44^. Additionally, adjacency effect correction is necessary to mitigate neighboring pixel interference. Moreover, Bidirectional Reflectance Distribution Function correction standardizes reflectance values across varying illumination and observation angles, and multiple scattering correction addresses atmospheric distortions. The resulting level 2 product included Bottom-of-Atmosphere reflectance with six spectral bands of 30 m spatial resolution for each image.

**Fig. 3:**
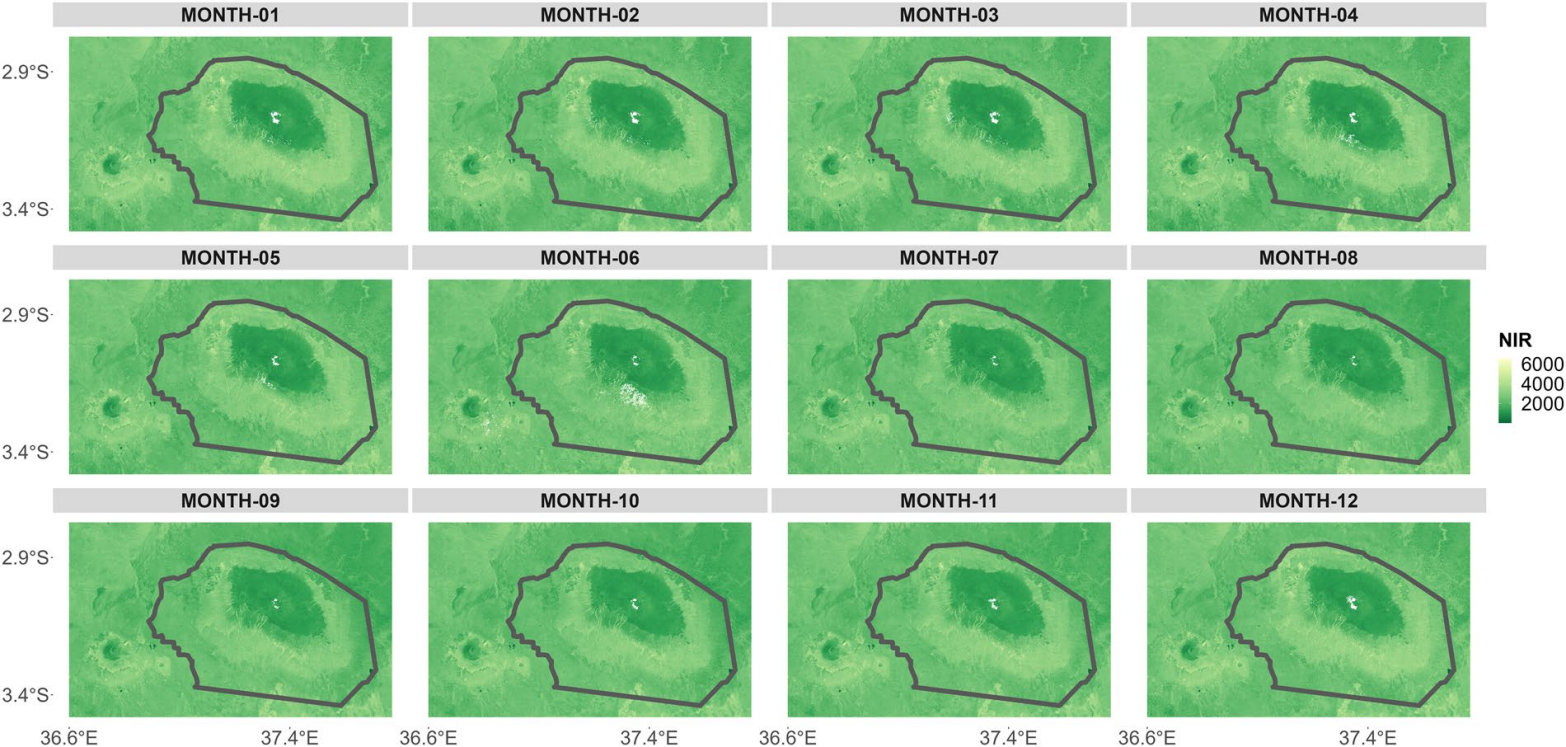
Base image: Visual representation of the Landsat base image time series. The base image time series consists of a monthly aggregate of all images from the near-infrared (NIR) channel. For this purpose, all images were averaged monthly over the years 2013 to 2021. This results in one image for each month, shown here as an example for the ecosystems of Mounts Kilimanjaro and Meru (outlined in black). The near-infrared values were scaled by 10,000.

The processed Landsat level 2 data were used to generate a “base image” (Figs. 2, 3). A base image is an interpolated time series data product with reduced gaps caused by non-equidistant earth observations^32^. All of the Landsat level 2 data from January 2013 to December 2021 were used to create the base image. This large dataset was necessary to achieve the most robust result possible, and using a large amount of data helps to minimize gaps caused by cloud cover. The base image was a level 3 time series product, in which near-infrared data of several years were aggregated by month. Our base image therefore consisted of 12 images - one for each month. In each monthly image, all the data from 2013 to 2021 for the respective month were aggregated to create one seamless, high-quality image that was subsequently used to align the Sentinel-2 images.

### Sentinel-2 level 2 processing with coregistration and level 3 processing

The Sentinel-2 data were processed to level 2 imagery (corresponds to ESA’s Sentinel-2 L2A data) using the same correction as applied for the Landsat level 2 processing. This included cloud detection and corrections including atmospheric correction, topographic correction, adjacency effect correction, Bidirectional Reflectance Distribution Function correction, and multiple scattering correction. During the *FORCE* level 2 processing, the Sentinel-2 data were geometrically aligned with the Landsat base image, a process referred to as “coregistration” by *FORCE*. Coregistration is important as it corrects the misalignment between Landsat and Sentinel-2 data, as well as improves the multitemporal inconsistency between the two Sentinel-2 satellites, i.e. Sentinel-2A and Sentinel-2B^31^. Correcting the alignment of the imagery can improve the overall accuracy of time series studies, for example, Rufin *et al*.^31^ found an average shift of images of 14 m in the x-direction and 13.4 m in the y-direction before coregistration and time series noise was effectively reduced by 43%. Moreover in level 2 processing the resolution of the 20 m Sentinel-2 bands was also enhanced to 10 m using “ImproPhe”, a data fusion method that predicted the 20 m resolution pixels while considering local pixel neighborhood at both resolutions, spectral distance, and multi-scale heterogeneity metrics^45^.

**Fig. 4:**
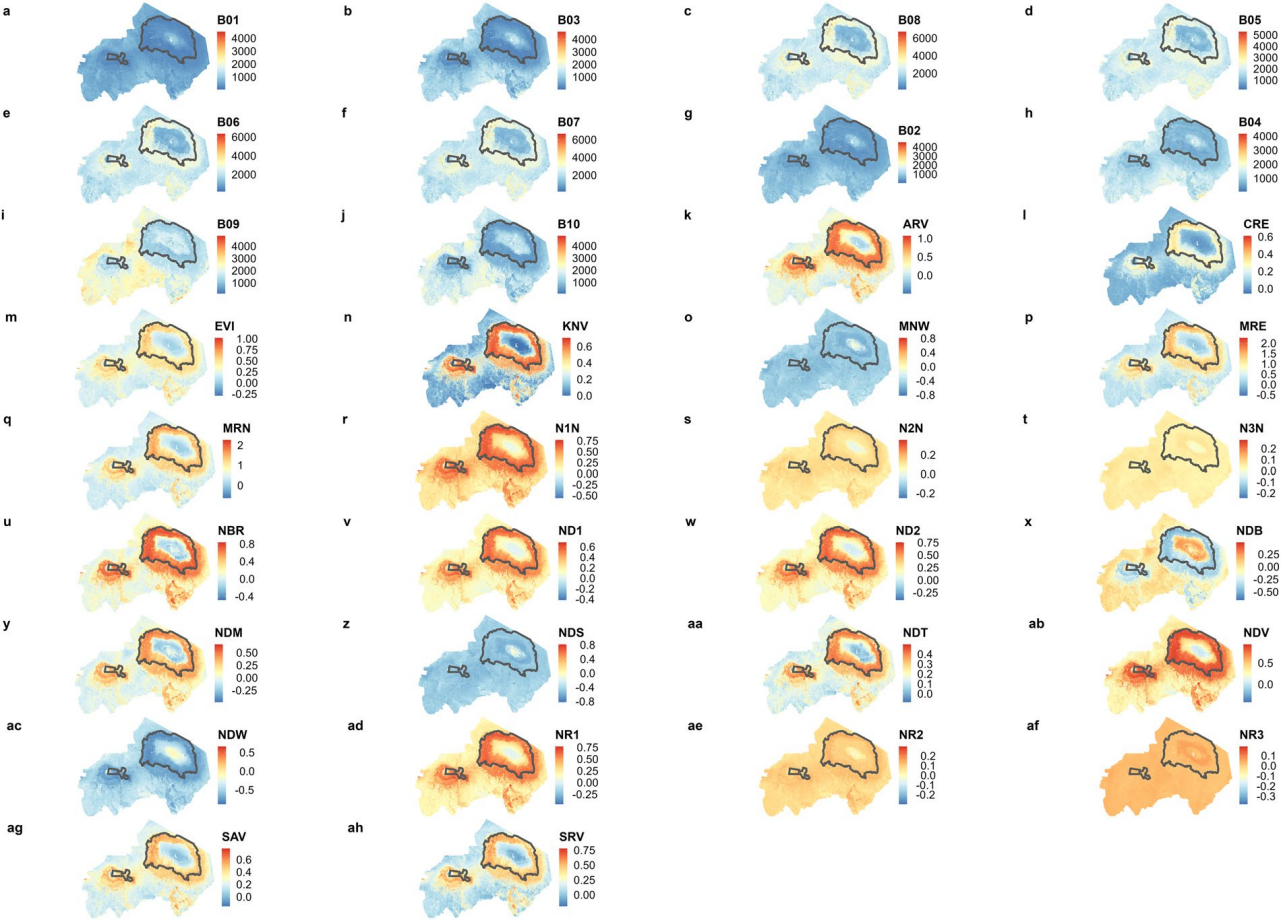
Spectral bands and indices: Representation of the spectral bands and indices provided in this dataset for Mounts Kilimanjaro and Meru for the year 2021. The abbreviations of the spectral bands and indices are listed in Table 1.

**Fig. 5:**
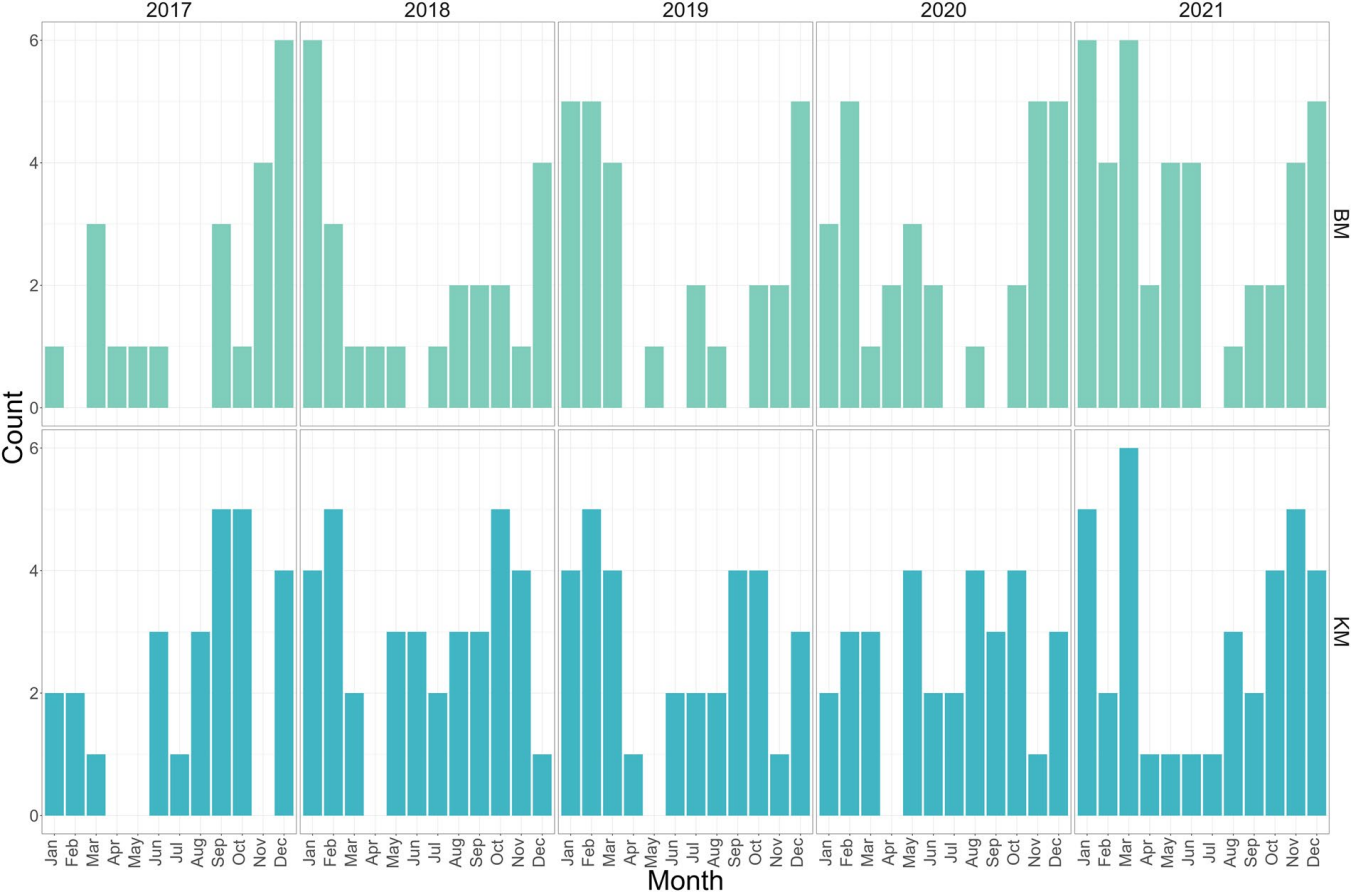
Monthly and yearly composite image counts. This figure displays the counts of B02 (blue) band images in a composite for each month and year, with the Bale Mountains (BM) depicted in the upper plot and Mounts Kilimanjaro and Meru (KM) in the lower plot. The y-axis represents the number of images for each month, while the x-axis corresponds to the months across the years 2017 to 2021.

The Sentinel-2 bands blue (B02), green (B03), red (B04), red edge 1 (B05), red edge 2 (B06), red edge 3 (B07), broad near-infrared (B08), near-infrared (B8A), short-wave infrared (B11) and short-wave infrared 2 (B12) are most commonly used in studies regarding landcover mapping^46^ and species distribution modeling^47^. Moreover, many studies use spectral temporal metrics to understand vegetation dynamics^48^, ecosystem disturbances (for example mapping burned areas^49^ and beetle outbreaks^50^), and assessing urban growth^51^. We processed the Sentinel-2 level 2 data (corresponds to ESA’s Sentinel-2 L2A data) to generate temporal aggregates as well as spectral temporal metrics^32^ (Fig. 2). Specifically the data were used to generate time series products with two different temporal resolutions. Two sets of images were created: one aggregated by year, resulting in one image for each year from 2017 to 2021, and another set aggregated by month, resulting in 12 images for each year from 2017 to 2021. For each image, in addition to the spectral bands, 24 spectral indices were calculated (Table 1; Fig. 4). The timely composites were created by computing the mean aggregate of all available images within the given time frame (e.g., month, year; Fig. 5). If due to excessive cloud cover no Sentinel-2 image for the time frame was available, an interpolation method was employed. The applied method involved radial basis function interpolation^36^, which considered data from 16 days before and 16 days after the target interpolation date.

**Table 1:** Abbreviations: Overview of satellite bands and spectral indices provided in the dataset.

Name	Abbreviation	Bandwidth/ formula
Blue band	B02	0.440–0.538 µm
Green band	B03	0.537–0.582 µm
Red band	B04	0.646–0.684 µm
Red edge 1 band	B05	0.694–0.713 µm
Red edge 2 band	B06	0.731–0.749 µm
Red edge 3 band	B07	0.769–0.797 µm
Broad near-infrared	B08	0.760–0.908 µm
Near-infrared band	B8A	0.848–0.881 µm
Short-wave infrared 1 band	B11	1.539–1.682 µm
Short-wave infrared 2 band	B12	2.078–2.320 µm
Atmospherically Resistant Vegetation Index^59^	ARV	(B8A - RB)/(B8A + RB) with RB = B04 - (B02 - B04)
Chlorophyll Index - Red Edge^60^	CRE	(B07/B05) - 1
Enhanced Vegetation Index^34^	EVI	G * ((B8A - B04)/(B8A + C1 * B04 – C2 * B02 + L)) with G = 2.5, L = 1, C1 = 6, C2 = 7.5
Kernel NDVI^61^	KNV	(1 - k)/(1 + k) with k = exp(-(B8A - B04)2/(2 * sigma2)) with sigma = 0.5 * (B8A + B04)
Modified Normalized Difference Water Index^62^	MNW	(B03 - B11)/(B03 + B11)
Modified Simple Ratio red edge^63^	MRE	((B08/B05) - 1)/sqrt((B08/B05) + 1)
Modified Simple Ratio red edge narrow^64^	MRN	((B8A/B05) - 1)/sqrt((B8A/B05) + 1)
Normalized Difference Vegetation Index red-edge 1 narrow^64^	N1N	(B8A - B05)/(B8A + B05)
Normalized Difference Vegetation Index red-edge 2 narrow^64^	N2N	(B8A - B06)/(B8A + B06)
Normalized Difference Vegetation Index red-edge 3 narrow^64^	N3N	(B8A - B07)/(B8A + B07)
Normalized Burn Ratio^65^	NBR	(B8A - B12)/(B8A + B12)
Normalized Difference Red Edge Index 1^66^	ND1	(B06 - B05)/(B06 + B05)
Normalized Difference Red Edge Index 2^67^	ND2	(B07 - B05)/(B07 + B05)
Normalized Difference Built-up Index^68^	NDB	(B11 - B8A)/(B11 + B8A)
Normalized Difference Moisture Index^69^	NDM	(B8A - B11)/(B8A + B11)
Normalized Difference Snow Index^70^	NDS	(B03 - B11)/(B03 + B11)
Normalized Difference Tillage Index^71^	NDT	(B11 - B12)/(B11 + B12)
Normalized Difference Vegetation Index^33^	NDV	(B8A - B04)/(B8A + B04)
Normalized Difference Water Index^35^	NDW	(B03 - B8A)/(B03 + B8A)
Normalized Difference Vegetation Index red-edge 1^66^	NR1	(B08 - B05)/(B08 + B05)
Normalized Difference Vegetation Index red-edge 2^64^61	NR2	(B08 - B06)/(B08 + B06)
Normalized Difference Vegetation Index red-edge 3^64^	NR3	(B08 - B07)/(B08 + B07)
Soil Adjusted Vegetation Index^72^	SAV	(B8A - B04)/(B8A + B04 + L) * (1 + L) with L = 0.5
Soil Adjusted and Atmospherically Resistant Vegetation Index^72^	SRV	(B8A - RB)/(B8A + RB + L) * (1 + L) with RB = B04 - (B02 - B04) with L = 0.5

The spectral resolution of Sentinel-2 bands was obtained from the ESA Sentinel-2 MultiSpectral Instrument user guide, available at https://sentinel.esa.int/web/sentinel/user-guides/sentinel-2-msi/resolutions/spectral.

### Rescaling and cropping

*FORCE* calculated the spectral indices as well as the bands (Table 1) not at their original scale, but inflated by a factor of 10,000. For example, the NDVI was not provided on its natural scale from −1 to 1 but as values from −10,000 to 10,000. To provide a user-friendly product, the images were rescaled to scales familiar to the users (Fig. 4). To rescale, we used the software R^52^ (version 4.2.1), together with the R package terra^53^ (version 1.5.34). Rescaling the spectral indices to familiar scales, like −1 to 1 for NDVI, is crucial for intuitive understanding and accurate analysis, ensuring ease of comparison and reducing the risk of misinterpretation among end users. In addition, all the images were cropped to the extent of the mountain ecosystem areas. While for Bale Mountains the whole Sentinel-2 tile T37NEH was used, the extent for Mounts Kilimanjaro and Meru are based on two region administrative boundaries Arusha and Kilimanjaro (it covers the districts Arusha, Arusha urban, Meru, Hai, Moshi, Moshi urban, Rombo and Siha). The boundaries were obtained via the humanitarian data exchange^54^.

## Data Records

Our data^55^ are accessible via the public repository data_UMR 10.17192/FDR/166 and consists of a workflow file and four parameter files, a python script, and a readme file as a text file as well as 10 .tar files containing the data. The data^55^ are identifiable by the mountain abbreviations e.g. Bale Mountains (BM) and Mounts Kilimanjaro and Meru (KM). All data^55^ files are in cloud optimized GeoTIFF format with SpatioTemporal Asset Catalogs (STAC) metadata as an additional .json file (one corresponding to each .tif file). We provide our data in ‘cloud optimized GeoTIFF’ format instead of the standard ‘TIFF’, as GeoTIFFs embed additional georeferencing details, coordinate systems, resolution, and information on the number of raster layers information, enhancing the utility and precision of the data. Cloud Optimized GeoTIFFs are also optimized for efficient streaming and access over the web, enabling users to quickly retrieve and analyze specific geographic information without downloading entire datasets. Each point in time (monthly and annual products) for each mountain ecosystem includes ten spectral bands (red, green, blue, broad near-infrared, near-infrared, red edge 1–3, and shortwave-infrared 1–2) as well as 24 calculated indices (Table 1). The total number of Sentinel-2 images for Bale Mountains, Mounts Kilimanjaro and Meru is 170 each for annual data, resulting in a total size of 87.9 GB (Bale Mountains) and 93.7 GB (Mounts Kilimanjaro and Meru). For monthly data, 170 twelve-layer images are included for each study area, with one layer per month, resulting in a size of 0.99 TB (Bale Mountains) and 1 TB (Mounts Kilimanjaro and Meru). There are 5 .tar files for Bale Mountains and 5 .tar files for Mounts Kilimanjaro and Meru containing the annual data (FBY). The .tar files with annual data can be directly downloaded from the repository. However, due to the substantial size of the monthly data, a download request must first be submitted to the repository, in order to download the monthly data.

The cloud optimized GeoTIFF files and .json files are named using the naming convention which can be seen in Table 2. Each string consists of the study area, its projection as EPSG code, sensor name, processing level, type of reflectance product, band name or spectral index, year, and the information if it is a monthly or yearly product. For example, an annual image of the year 2017 for the NDVI for the Bale Mountains is named like this: BM_EPSG32637_SEN2_L3_BOA_NDV_2017_FBY.tif. The .json files follow the same naming convention as the cloud optimized GeoTIFF files. The analysis-ready satellite images for the Bale Mountains are in WGS84 UTM zone 37N and in UTM zone 37S for Mounts Kilimanjaro and Meru. The dataset^55^ contains a workflow.txt file, which includes all the commands used within *FORCE* for downloading and processing the satellite images. Additionally, it provides all the parameter settings used in this study in the order of their usage: landsat_level2, ls_base, sentinel_level2, and sentinel_level3. These files are supplied in .prm format. The STAC metadata was created using the Python script generate_stac_metadata_from_cogtif.py.

**Table 2:** Naming convention used for all Sentinel-2 data provided in this study.

Digits	Description
1–2	Name of the study area. Either Mounts Kilimanjaro and Meru (KM) or Bale Mountains (BM)
4–12	Projection used: either EPSG32737 for Mounts Kilimanjaro and Meru or EPSG32637 for Bale Mountains
14–17	Sensor name. Sentinel-2 for all available images
19–20	Processing level
22–24	Bottom of the Atmosphere (BOA) product
26–28	Either Sentinel-2 band name or name of the spectral index (for the full list of abbreviations see Table 1)
30–33	Year
35–37	Indication if the image was folded by month (FBM) or year (FBY)
38–42	File type GeoTIFF or .json

## Technical Validation

**Fig. 6:**
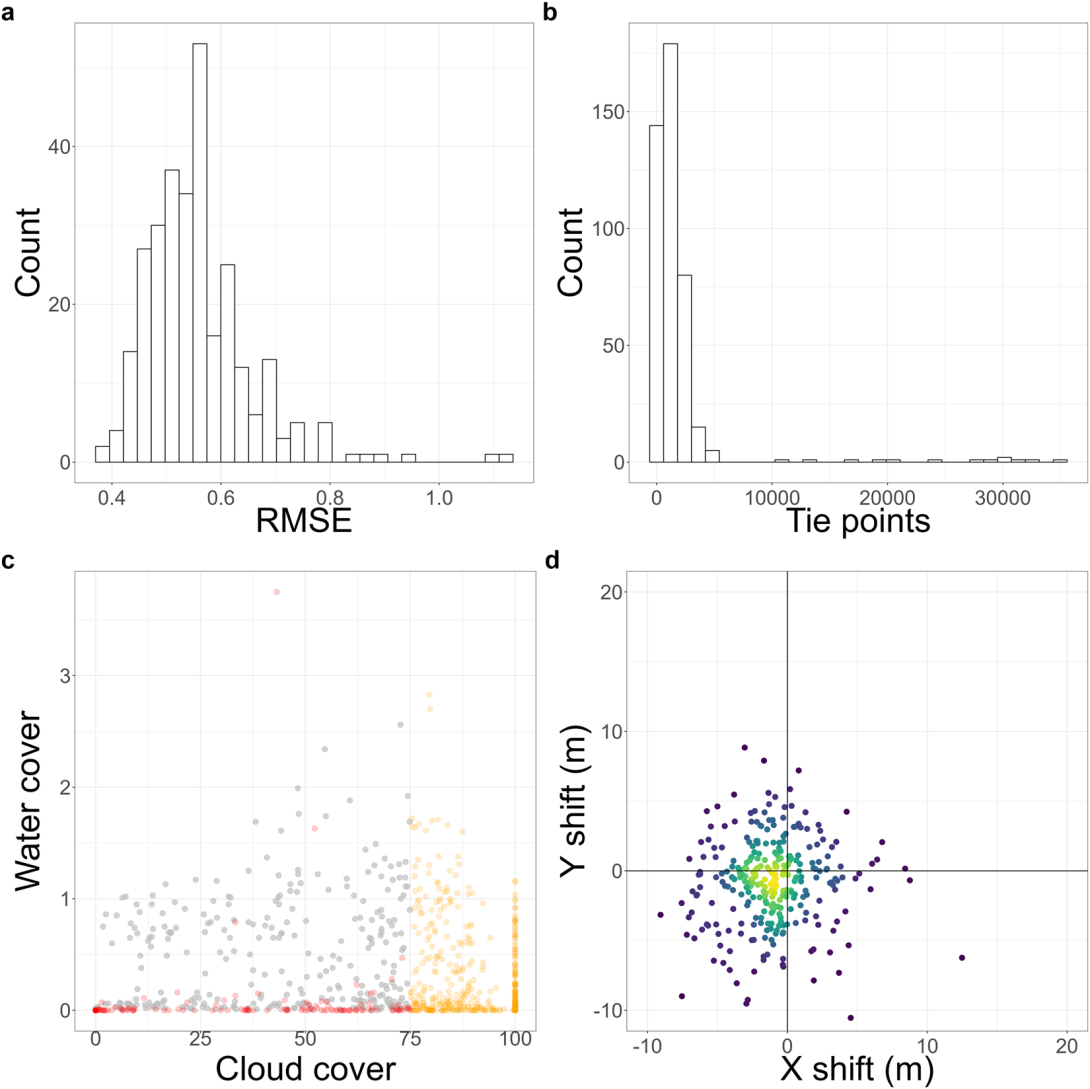
Mounts Kilimanjaro and Meru geometric alignment. (**a**) Root mean square error (RMSE) of the coregistered images (x-axis) and number of images with the corresponding RMSE (y-axis). (**b**) Number of tie points detected per image (x-axis) and count of images with the corresponding number of tie points (y-axis). (**c**) Percentage of water (y-axis) and cloud cover (x-axis) for all images. Orange points represent processing failures due to excessive cloud cover, red points indicate coregistration failures, and grey points signify successful processing. (**d**) Image shift in meters performed during coregistration.

Our dataset^55^ has undergone the default technical validation steps implemented in *FORCE*. This guarantees a very high quality of the datasets, as datasets that are flawed, have too much cloud cover, or in which the coregistration failed are automatically sorted out. We applied coregistration on 972 images for Mounts Kilimanjaro and Meru, out of which 292 were successfully coregistered, while coregistration failed for 144 images, 29 showed error, and 507 images were categorized as too cloudy. The mean RMSE of the coregistration was 0.56 (Fig. 6a). Figure 6b shows the number of tie points used in coregistration and Fig. 6c shows images where the cloud cover was too high, these images were discarded from further processing. There was an average shift in images of −1.11 m (standard deviation: 2.9 m) and −0.83 m (standard deviation: 3.1 m) in the x- and y-direction respectively. The maximum shift in x-direction was 12.49 m and y-direction was 8.84 m (Fig. 6d).

**Fig. 7:**
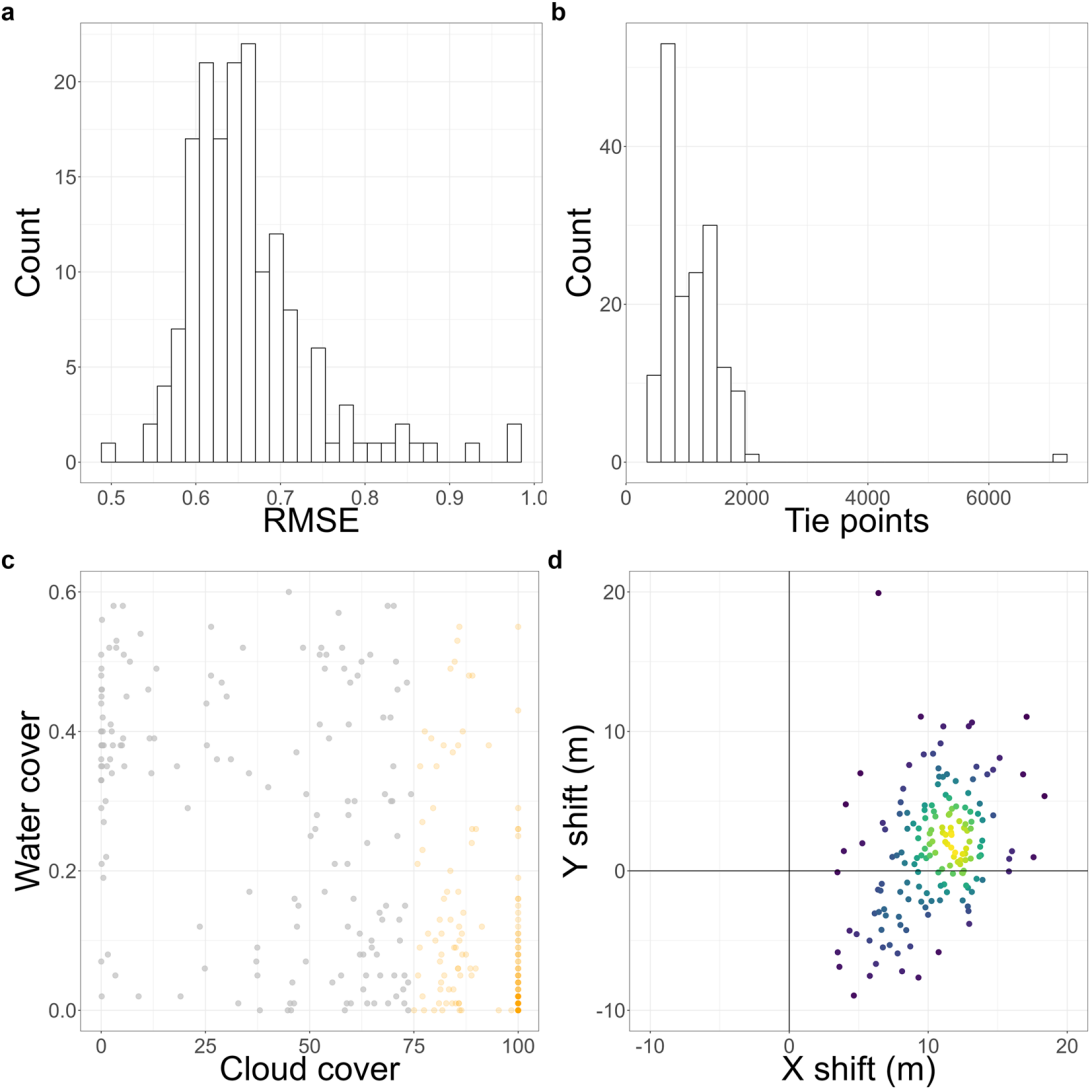
Bale Mountains geometric alignment. (**a**) Root mean square error (RMSE) of the coregistered images (x-axis) and number of images with the corresponding RMSE (y-axis). (**b**) Number of tie points detected per image (x-axis) and count of images with the corresponding number of tie points (y-axis). (**c**) Percentage of water (y-axis) and cloud cover (x-axis) for all images. Orange points represent processing failures due to excessive cloud cover, red points indicate coregistration failures, and grey points signify successful processing. (**d**) Image shift in meters performed during coregistration.

Similarly for the Bale Mountains 364 Sentinel-2 images were used for coregistration out of which 162 were coregistered successfully and 202 images were categorized as too cloudy. The mean RMSE of the coregistration was 0.67 (Fig. 7a). Figure 7b shows the number of tie points used in coregistration and Fig. 7c shows images where the cloud cover was too high, these images were also discarded from further processing. Furthermore, there was a mean shift of 10.48 m (standard deviation: 2.9 m), and 1.56 m (standard deviation: 4.3 m) in the x- and y-direction, respectively (Fig. 7d). The maximum image shifts were 18.37 m in the x-direction and 19.92 m in the y-direction.

**Fig. 8:**
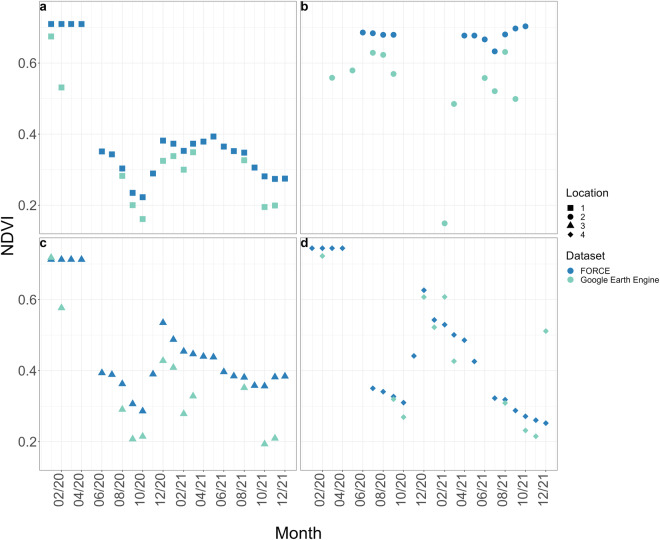
Comparative analysis of *FORCE* processed Normalized Difference Vegetation Index (NDVI) vs. NDVI derived from Sentinel-2 L2A data obtained via Google Earth Engine. This figure presents a comparison between the NDVI values processed by *FORCE* and those derived from Sentinel-2 L2A data using Google Earth Engine, focusing on the years 2020 and 2021. The NDVI values (y-axis) are plotted against time (x-axis), and each of the four plots represents a distinct point of interest within the Mounts Kilimanjaro and Meru ecosystem.

Furthermore, the quality of our dataset was compared with a dataset derived from cloud-ready-to-use platforms (such as Google Earth Engine). We chose NDVI for this comparison because it is a common spectral index for assessing vegetation health, monitoring environmental changes, and supporting studies in urban planning, disaster response, and biodiversity conservation^56^. We compared the NDVI values derived from our dataset^55^ processed with *FORCE* and a harmonized Sentinel-2 dataset (COPERNICUS/S2_SR_HARMONIZED) derived via Google Earth Engine^57^. Fig. 8 shows the mean NDVI values for four points in the Mounts Kilimanjaro and Meru ecosystems derived using *FORCE* and Google Earth Engine. While for some points (e.g., point 1) both datasets seem quite consistent, for others the *FORCE* dataset provided a smoother time series.

## Usage Notes

The dataset^55^ consists of cloud optimized GeoTIFF files which can be opened and analyzed further by using a Geographic Information System software, for example, the freely available software QGIS^58^ or a script based programming language for data analysis such as R^52^ or Python. With R the files can be opened and processed with the R package terra^53^. For efficient data retrieval from the repository, we recommend employing the command line utility wget (https://www.gnu.org/software/wget/).

## Data Availability

The code used to process the Sentinel-2 data with *FORCE* version 3.7.10 can be obtained via data_UMR 10.17192/FDR/166.
